# Selection of the Best Electroencephalogram Channel to Predict the Depth of Anesthesia

**DOI:** 10.3389/fncom.2019.00065

**Published:** 2019-10-01

**Authors:** Clement Dubost, Pierre Humbert, Arno Benizri, Jean-Pierre Tourtier, Nicolas Vayatis, Pierre-Paul Vidal

**Affiliations:** ^1^Department of Anesthesiology and Intensive Care, Begin Military Hospital, Saint-Mande, France; ^2^Cognac-G Cognition and Action Group, CNRS, Université Paris Descartes, SSA, Paris, France; ^3^Centre de Mathematiques et de Leurs Applications, CNRS, ENS Paris-Saclay, Université Paris-Saclay, Cachan, France; ^4^Institute of Information and Control, Hangzhou Dianzi University, Zhejiang, China

**Keywords:** consciousness, general anesthesia, electroencephalography, depth of anesthesia, machine learning, brain monitoring

## Abstract

Precise cerebral dynamics of action of the anesthetics are a challenge for neuroscientists. This explains why there is no gold standard for monitoring the Depth of Anesthesia (DoA) and why experimental studies may use several electroencephalogram (EEG) channels, ranging from 2 to 128 EEG-channels. Our study aimed at finding the scalp area providing valuable information about brain activity under general anesthesia (GA) to select the more optimal EEG channel to characterized the DoA. We included 30 patients undergoing elective, minor surgery under GA and used a 32-channel EEG to record their electrical brain activity. In addition, we recorded their physiological parameters and the BIS monitor. Each individual EEG channel data were processed to test their ability to differentiate awake from asleep states. Due to strict quality criteria adopted for the EEG data and the difficulties of the real-life setting of the study, only 8 patients recordings were taken into consideration in the final analysis. Using 2 classification algorithms, we identified the optimal channels to discriminate between asleep and awake states: the frontal and temporal F8 and T7 were retrieved as being the two bests channels to monitor DoA. Then, using only data from the F8 channel, we tried to minimize the number of features required to discriminate between the awake and asleep state. The best algorithm turned out to be the Gaussian Naïve Bayes (GNB) requiring only 5 features (Area Under the ROC Curve - AUC- of 0.93 ± 0.04). This finding may pave the way to improve the assessment of DoA by combining one EEG channel recordings with a multimodal physiological monitoring of the brain state under GA. Further work is needed to see if these results may be valid to asses the depth of sedation in ICU.

## 1. Introduction

An optimal General Anesthesia (GA) state is assessed by the Depth of Anesthesia (DoA) defined by the experts as “the probability of non-response to stimulation, calibrated against the strength of the stimulus, the difficulty of suppressing the response, and the drug-induced probability of non responsiveness at defined effect site concentrations” (Shafer and Stanski, 2008). Ensuring an adequate DoA is important to avoid awareness during the surgery without overdosage of anesthetics drugs. In 2014, the 5th National Audit Project (NAP5) estimated the incidence of Accidental Awareness during General Anesthesia (AAGA), to be roughly 1/19,000 (Pandit et al., 2014). It is important to detect such event because it exposes the patient to complications like post-operative stress disorder (Pandit et al., 2014) or postoperative delirium (Fritz et al., 2016). An ideal DoA monitor should exhibit high sensitivity, specificity, and positive predictive values as a diagnostic tool. Moreover, its output should relate to the probability of consciousness in real time and should fulfill practical criteria such as being portable, easy to use, cost-effective and safe (Pandit et al., 2014). Such a monitor providing a precise estimation of the DoA remains an unmet need so far.

Currently, DoA is assessed by calculating appropriate drug concentration for each patient and monitoring physiological variables such as heart rate, blood pressure, oxygen concentration, eye lash reflex, patient reactions and Minimal Alveolar Concentration (MAC), which is the concentration of inhaled anesthetic required to suppress movement to a surgical incision in 50% of the patients (Campagna et al., 2003). While these variables give valuable information, ElectroEncephaloGram (EEG) should also be an obvious candidate to monitor DoA because the brain is the targeted organ of anesthesia. However, it is only used in between 2.8 and 4.3% of anesthesia in a clinical setting (Pandit et al., 2014). This is due to the numerous limitations of the available EEG-based DoA monitors (Bruhn et al., 2006). In particular to know which EEG channels would be optimal to monitor anesthesia remains elusive. While several studies and reviews focused on the question of EEG channel reduction (Al-Ani and Al-Sukker, 2006; Arvaneh et al., 2011; Alotaiby et al., 2015) in the context of the control of sleep and wakefulness or motor imagery, they remain scarce in the field of GA. Different pairs of channels had been used to monitor the DoA, mainly located on the frontal and temporal locations [F3-T3 and F4-T4 (Khan et al., 2014), Fp1-Fp2 (Sleigh et al., 2001), AT1, M2, Fz (Schneider et al., 2014)], and it is also the case for the Bispectral (BIS) and Entropy monitors. However, to the best of our knowledge, a study investigating which to the best of our knowledge, a study investigating which subset of EEG channels should be preferentially used to monitor the DoA remains to be done.

Over the last several years, the use of Machine Learning (ML) in neuroscience has been rapidly increasing with a wide range of applications. For instance, recent advances in computer vision using ML are becoming important tools for image-based cancer detection (Hu et al., 2018). Brain-computer interface or close-loop control are also two important areas of research in neuroscience and anesthesia (Dumont, 2012). Another major use of ML in neuroscience is to examine which input variables allows better understanding of the behavior of the brain and the relationship between its areas. There are many methods to establish this feature selection. One of the simplest methods, known as Wrappers, takes advantage of the learning performance of a classifier to assess the quality of selected features (Li et al., 2018). This is the method used in this paper.

In neuroscience, these features most often come from brain EEG signals. These laters are widely studied in ML to improve different tasks such as age prediction (Al Zoubi et al., 2018), EEG classifications and features extractions (Amin et al., 2017; Wang and Veluvolu, 2017). More related to our work, recently Bresch et al. have studied the use of deep neural network for real-time sleep stage classification from single channel EEG (Bresch et al., 2018).

In this paper, we investigated this question by monitoring during GA the physiological variables mentioned above together with the brain activity using 32-channel EEG. Then, we employed a ML-based approach, to determine the optimal EEG channel to assess the DoA.

## 2. Materials and Methods

### 2.1. Study Design

This was a single-center observational study of patients undergoing GA with propofol/sufentanil induction and maintenance by sevoflurane. Patients were included if they were scheduled for an outgoing surgery under GA in the Begin military hospital between March and May 2018. The study protocol has been approved by Pr. JE Bazin, head of the ethics committee of the French society of anesthesiology (SFAR) under the number IRB 00010254-2016-018, and the protocol was in accordance with the Declaration of Helsinki.

### 2.2. Inclusion and Exclusion Criteria

The patients included in this study were (1) American Society of Anesthesia I–III patients (ASA Physical Status Classification System. American Society of Anesthesiologists; https://www.asahq.org/resources/clinical-information/asa-physical-statusclassification-system) (2) between 18 and 80 years old, and (3) gave their written informed consent to the study. Patients were excluded if they presented complications during the surgery (cardiac arrhythmias, variation of the blood pressure or cardiac frequency more than 20% compared to the baseline value, or unplanned hospitalization).

### 2.3. Anesthesia Protocol

All patients were pre-oxygenated via face-mask by 100% oxygen for at least 3 min before induction. Sufentanil 0.3 μg/kg of ideal-body weight was injected rapidly followed, 3 min later, by 2–4 mg/kg of propofol in combination with ketamine 20 mg. When required for the surgery, patients were paralyzed following induction with a bolus of 0.17 mg/kg of cisatracurium. After tracheal intubation, patients were ventilated with tidal volume of 6 mg/kg ideal-body weight, 5 *cmH*_2_*O* Positive end-expiratory pressure (Peep) and a respiratory rate between 10 and 14/min to maintain *EtCO*_2_ between 30 and 40 mmHg. Anesthesia was maintained with sevoflurane MAC age-adjusted (e.g., 1.0). Dose adjustments were made by the anesthesiologist in charge of the patient depending on clinical variables available. Once asleep, patients received a single bolus of local anesthesia when indicated for the surgery.

### 2.4. Clinical Assessment of Consciousness

During the present study, the DoA was continuously assessed by the anesthesiologist or by the nurse anesthesiologist, using standard tools and unit protocols. Adequate anesthesia was defined as a patient having received the anesthetic drugs, being unresponsive to stimulus and to surgery, with a MAC above 0.7. Arousal was defined as a patient being responsive to simple orders. The time points of propofol administration, loss of consciousness assessed clinically (i.e., corresponding to loss of verbal contact and response to simple order, respectively), tracheal intubation, and return of consciousness were recorded. The DoA was continuously monitored both clinically and thanks to the Bispectral Index (BIS).

### 2.5. Monitoring and Data Collecting

During the surgery, patients were continuously monitored with a multiparametric device, the *Carescape monitor B850*, from General Electrics (GE) Healthcare™ Finland Oy, Helsinki, Finland. The monitoring included electrocardiogram (EKG), arterial pulse oximetry, non-invasive arterial blood pressure, gas analysis and plethysmography. The following variables were recorded with a sampling frequency of 1 *Hz* during the anesthesia: systolic, diastolic and mean arterial blood pressures, arterial pulse oximetry, end-tidal *CO*_2_, MAC, inspired fraction of sevoflurane, end-tidal sevoflurane, BIS (Quatro Sensor manufactured by Covidien), and inspired fraction of *O*_2_. The heart rate and heart derivation Lead II were recorded at 300 Hz. All the recorded variables are presented in the Table 1.

**Table 1:** List of the variables recorded and their respective frequencies.

**Variable**	**Abbreviation**	**Frequency of recording**	**Only during GA**
Electroencephalogram	EEG	250 Hz	No
Electrocardiogram Lead II	EKG	300 Hz	No
Heart rate	HR	1 Hz	No
Systolic arterial blood pressure	SBP	1 Hz	No
Diastolic arterial blood pressure	DBP	1 Hz	No
Mean arterial blood pressure	MBP	1 Hz	No
Pulse oxymetry	*SpO*_2_	1 Hz	No
End tidal carbon dioxyde	*EtCO*_2_	1 Hz	Yes
Mean alveolar concentration	MAC	1 Hz	Yes
Fraction inspired sevoflurane	*FiSevo*	1 Hz	Yes
End tidal sevoflurane	*EtSevo*	1 Hz	Yes
Fraction inspired of dioxygen	*FiO*_2_	1 Hz	Yes
Respiratory rate	RR	1 Hz	Yes
Bispectral index	BIS	1 Hz	Yes

We used SmartRea Monitor, a personal software developed by our lab. During the surgery, the following time-stamps were noted precisely: beginning of the anesthesia (with the different drugs injected), loss of eyelash reflex, mechanical ventilation, intubation of the trachea, beginning of the surgery, end of surgery, answer to basic verbal command (grasping the hand or opening the eyes on command) and tracheal extubation. The monitoring from the multiparametric device was stopped just before leaving the Operating Room (OR) for the Post-Anesthesia Care Unit (PACU) and was then restarted until the patient was transferred to the out-patient ward.

### 2.6. EEG Acquisition

**Figure 1:**
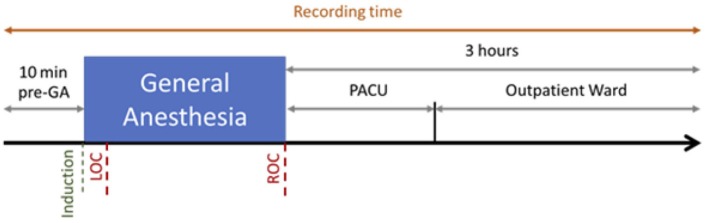
Recording Session for one patient. The recording began 10 min before induction of propofol and Loss Of Consciousness (LOC), and lasted 3 h after the Recovery Of Consciousness (ROC), including 1 h in the Post-Anesthesia Care Unit (PACU) and 2 h in the Outpatient Ward.

EEG was recorded using a Brain Vision actiCHamp amplifier with 32 active electrodes. The cap was put on the patient in their room, and a SuperVisc Electrolyte-Gel was applied immediately after. The electrodes were placed following the standard 10–20 system. The reference and the mass were located at Fz and Fpz, respectively. The EEG electrodes were protected by a disposable cap. We waited until the electrodes reached an impedance between 20 and 400 ohms. Electrodes with impedance over 500 *ohms* throughout the recording were ignored. The EEG signal was recorded at 250 Hz–24 bits thanks to the same software than for the monitoring (SmartRea). The recording began 10 min before inducing the anesthesia and lasted until 3 h after arousal (Figure 1).

### 2.7. EEG Data Processing

#### 2.7.1. Preprocessing

**Figure 2:**
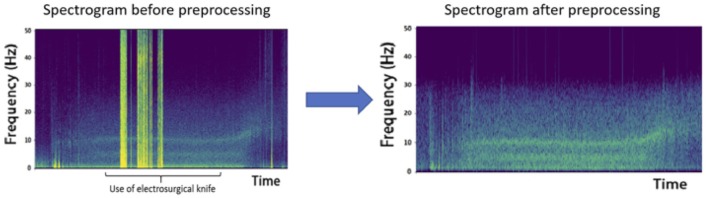
Effect of electrosurgical knife electromagnetic emissions on the recordings. The high amplitude epochs obtained are due to these emissions and represent a heavy artifact that must be removed from analysis, as shown after preprocessing.

The signal was digitally filtered using a Butterworth bandpass filter of order 5 between 1 and 30 Hz to remove the potential drift below 1 Hz, to keep the frequencies characterizing GA and to remove any noise over 30 Hz. The spectrogram of each channel in every recording has been plotted and visually inspected by a couple of a neuroscience engineer and an experienced anesthesiologist. A channel was considered unexploitable when more than 30% of the recording presented artifacts. If too many channels of a recording were classified unexploitable, the patient's data was excluded from the analysis. If only a few channels were considered bad, they were marked as such and were excluded from analysis. The data was segmented into epochs of 2 s. To remove obvious artifacts such as electrosurgical knife emissions (Figure 2) or brutal head movements typically characterized by a high amplitude (Nolan et al., 2010), we marked as bad every epoch with an amplitude higher than 0.3 mV.

Independent Component Analysis (ICA) (Makeig et al., 1996) on each recording was used to remove vertical and horizontal ElectroOculoGraphic (EOG) artifacts. ICA is a multivariate method used in several fields such as neuroscience (Makeig et al., 1996; Calhoun et al., 2001; Beckmann and Smith, 2004), or biology (Lee and Batzoglou, 2003; Scholz et al., 2004). One underlying assumption of ICA is that the data are a combination of latent components which are statistically independent. In particular, linear ICA problem addresses the case where latent variables and observations are linked by a linear transformation. Hence, in linear ICA we want to estimate a linear transformation of the input signals into “source signals” which are as independent as possible.

More formally, given *N* signals *x*_1_, ··· , *x*_*N*_ of length *T* arranged in a matrix *X*, linear ICA is based on the model *X* = *AS* where *A* is the unknown mixing matrix, and *S* is the source matrix with *N* statistically independent zero-mean rows. For ICA to become a well-posed problem it has been proved that we only required that all sources except one are non-Gaussian and statistically independent (Comon, 1994). The challenge is to recover *A* and *S*.

The ICA algorithm is therefore effective for source separation task where (1) the mixing is linear and propagation delays negligible, (2) the time courses of the sources are independent, and (3) the number of sources is the same as the number of sensors.

In the case of EEG, neural activity is instantaneously and linearly spread across channels, due to Maxwell's equations (Hari and Puce, 2017), hence assumption (1) holds. Assumption (2) is reasonable because the most commun artifacts (eye and muscle activity, cardiac signals) are independant from the activity of cortical neurons. Assumption (3) remains unknown since we do not know the effective number of statistically-independent signals contributing to the EEG signals. Fortunately, numerical simulations have confirmed that the ICA algorithm can accurately identify the time courses of activation and the scalp topographies.

**Figure 3:**
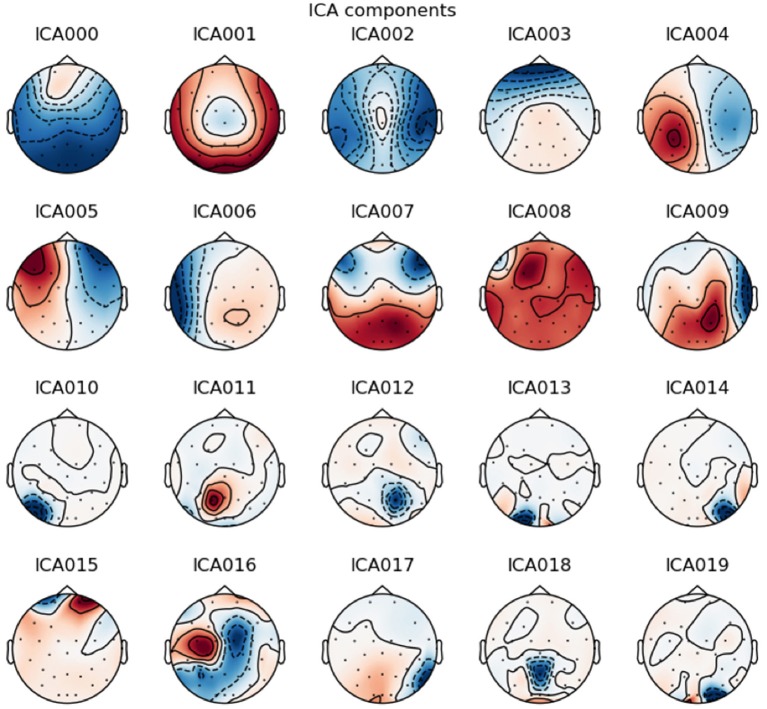
Example of an Independent Component Analysis on one patient. The component 3 is typically vertical eye blink due to the frontal correlated location of the component. The component 5 is typically horizontal eye movement due to the frontal anticorrelation location around each eye. These components have been selected for removal.

**Figure 4:**
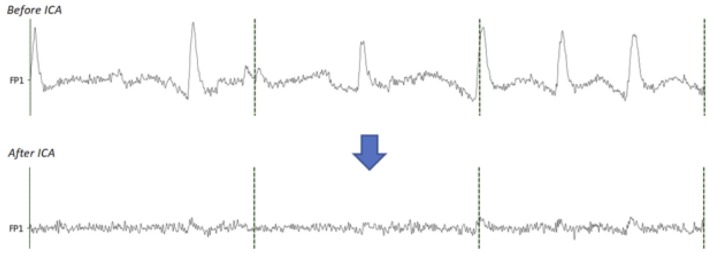
Comparison between before and after the EOG components removal with ICA. On the signal before ICA, the eye blinks are clearly visible. On the signal after removing the EOG components, the eye blinks are extremely reduced.

In order to perform ICA, we used the ICA algorithm implemented in the python library MNE. EOG and heart rate artifacts were removed and the data were then recreated without these components (Figures 3, 4).

**Figure 5:**
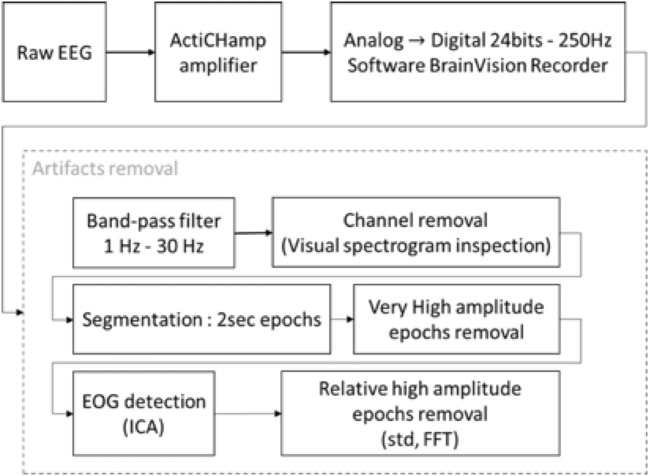
Block diagram of EEG recording and processing. The raw signal was recorded with an ActiChamp amplifier in 24 bits–250 Hz. Due to the lengths of the recordings and the large number of epochs, the artifact removal was partially unsupervised. The recordings were filtered with a BPF between 1–30 Hz. The spectrograms of the channels were plotted for visual inspection, bad channels were marked and excluded from the analysis. An Independent Component Analysis (ICA) was used to remove Electrooculographic (EOG) artifacts. Each epoch was compared to various threshold on their time series and power spectrum and were kept or rejected depending of these thresholds.

Small head movements may remain undetected by the previous procedure but still contaminate some epochs. To detect those artifacts, we calculated for each channel the standard deviation in three different states: Awake before GA, Asleep, and Awake after GA. Criteria defining an appropriate DoA and arousal have been stated previously (see section 2.4). We then applied an amplitude threshold for each epoch of these states. We often found artifacts resembling very regular sinusoidal waves with high amplitude. To detect these artifacts, we calculated the Fast Fourier Transform (FFT) of the epoch and compared the maximal amplitude with the standard deviation of the FFT, thus detecting relative high amplitude on single frequencies which would be the case for a sine wave. All the steps of the preprocessing are summarized in the Figure 5.

#### 2.7.2. Processing

After preprocessing, our dataset contained 9,630 epochs labeled “awake” and 12,665 epochs labeled “asleep.” This represents 43% of awake epochs and 57% of asleep epochs. We considered the labels “asleep” as positive and “awake” as negative. To differentiate awake state from sleeping state, we calculated 10 features, based on the repartition of delta, theta, alpha and beta powers in the EEG signals, on each epoch of every channels and for all the subjects after preprocessing (Table 2).

**Table 2:** List of the features calculated on the dataset.

**Nb**	**Features**	**Description**
1	Standard deviation	Standard deviation in the time domain
2	Sample Entropy	Approximate sample entropy of the epoch
3	Mean Power Spectrum	Mean value of the power spectrum from 0 to 30 Hz
4	Power Spectrum Delta	Mean value of the power spectrum of Delta frequencies (0–4 Hz)
5	Power Spectrum Theta	Mean value of the power spectrum of Delta frequencies (4–8 Hz)
6	Power Spectrum Alpha	Mean value of the power spectrum of Delta frequencies (8–12 Hz)
7	Power Spectrum Beta	Mean value of the power spectrum of High Beta frequencies (18–30 Hz)
8	Ratio PWS Delta/Beta	Ratio of the mean Power Spectrum Delta frequencies/Beta frequencies
9	Ratio PWS Theta/Beta	Ratio of the mean Power Spectrum Theta frequencies/Beta frequencies
10	Ratio PWS Alpha/Beta	Ratio of the mean Power Spectrum Alpha frequencies/Beta frequencies

We used features of Table 2 to train the following ML algorithms:

K-Nearest Neighbors (KNN): the output label is based on the k closest training examples from the feature space.

Decision Tree Learning (DTL): recursive algorithm treating the feature space with a decision tree where each internal node corresponds to a feature and each leaf corresponds to a label.

Quadratic Discriminant Analysis (QDA): draws a quadratic boundary between the dataset to maximize the separation of the class labels.

Let consider some data (*X, y*), where *X* are observations and *y* is the class variable.

QDA is derived from probabilistic models which model the class conditional distribution of the data ℙ(*X* ∣ *y* = *k*) for each class *k*. Prediction is then obtain using the bayes formula

ℙ(y=k|X)=ℙ(X∣y=k)ℙ(y=k)/ℙ(X)

and by selecting the class *k* which maximizes this conditional probability. In QDA, ℙ(*X* ∣ *y*) is modeled as a multivariate gaussian distribution. Notice that, in the case of QDA, there are no assumptions on the covariance matrices of the Gaussians. In order to perform QDA, we used the QuadraticDiscriminantAnalysis method implemented in the python library sklearn.

Gaussian Naive Bayes (GNB): classification based on the assumption that the value of a particular feature in a class is independent of the value of any other feature, which is why it is called “naive.”

Given a class variable *y* and feature vectors *x*_1_, ··· , *x*_*N*_,

$$\mathbb{P}\left(y|x_{1},\cdot \cdot \cdot \;,x_{N}\right)=\mathbb{P}\left(y\right)\mathbb{P}\left(x_{1},\cdot \cdot \cdot \;,x_{N}|y\right)/\mathbb{P}\left(x_{1},\cdot \cdot \cdot \;,x_{N}\right).$$

Using the naive conditional independence assumption

$$\mathbb{P}\left(y||x_{1},\cdot \cdot \cdot \;,x_{N}\right)=\mathbb{P}\left(y\right)\overset{N}{\underset{i=1}{\prod }}\mathbb{P}\left(x_{i}|y\right)/\mathbb{P}\left(x_{1},\cdot \cdot \cdot \;,x_{N}\right).$$

The classification rule is given by

$$\arg\underset{y}{\max}\mathbb{P}\left(y\right)\overset{N}{\underset{i=1}{\prod }}\mathbb{P}\left(x_{i}|y\right).$$

In Gaussian Naive Bayes, the likelihood of the features is assumed to be Gaussian. In order to perform GNB, we used the GaussianNB method implemented in the python library sklearn.

The last two algorithms were used for channel selection (QDA, GNB) whereas the four algorithms were used for features selection. We used the Wrappers method, one instance of subset selection techniques (Li et al., 2018).

#### 2.7.3. Channel Selection

Considering each channel individually, we computed the previously defined features (see Table 2). With these features, we used QDA and GNB algorithms to classify states between Awake and Asleep. These two algorithms do not need hyperparameters which make them suitable for small dataset. Before any computation, we separated our dataset multiple times in 3 subsets: Training, Validation, and Test sets. We used a leave-p-out procedure with *p* = 2. Hence, all possible subsets were tried for a total of 168 tries with 5 patients in the Training set, 2 in the Validation set, and 1 in the Test set. The Training set was used to train the algorithm. The Validation set was used to compute the Area Under the Curve (AUC) of each channel. The numerical integration of the AUC was calculated by the trapezoid method. We then selectionned the channels with the best mean AUC with the smallest standard deviation on these Valid tests. Finally, the Test set was used to expose the AUC of an unknown subject based on the results obtained with the Valid set.

## 3. Results

**Figure 6:**
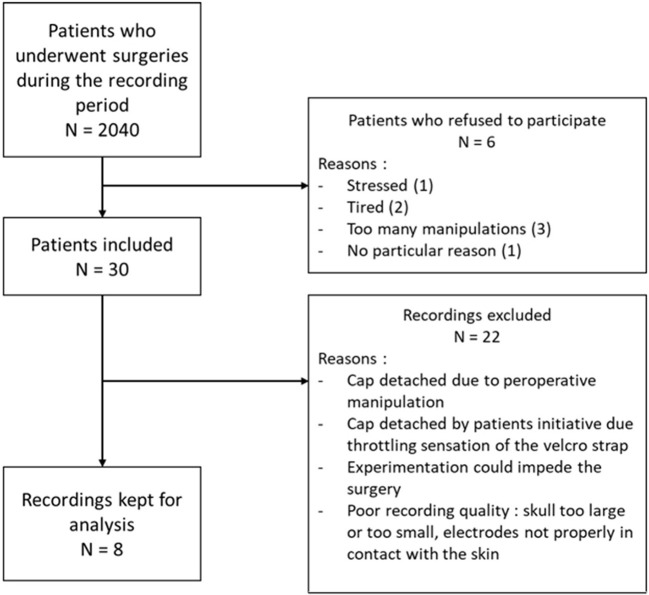
Flow chart of the inclusions with the number of patients operated in the period of the inclusions, the number of patients included, the number of patients kept for analysis and the main reasons of exclusion.

**Figure 7:**
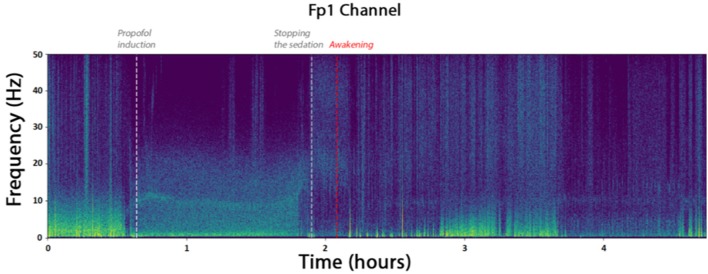
Spectrogram of the channel FP1 of the patient. The times marked as “propofol induction,” “stopping the sedation,” and “Awakening” are reported from time marks written down during the surgery. They coincide with the apparition of alpha waves and reduced beta waves.

From February to May 2018, 30 patients have been included. Demographic and epidemiologic data are presented in Table 3. The flow chart of the inclusions is presented in Figure 6. Due to the need of a high quality EEG in the remote environment of surgical theater, we kept only 8 patients for the final analysis. To obtain quick visual inspections, we drew the raw spectrograms of every channel for each recording before any preprocessing. On the spectrogram Figure 7, we notice drastic changes in the brain wave oscillations during GA, which are the clear apparition of alpha waves and the reduction of frequencies over 20 Hz. These particularities disappeared at the end of the anesthesia.

**Table 3:** Patients metadata containing their range of age, weight, height, surgery underwent and ASA score.

**Patient nb**	**Age**	**Weight (kg)**	**Height (cm)**	**Surgery**	**ASA**
1	50–60	115	196	Umbilical hernia repair	1
2	60–70	70	169	Prostate resection	3
3	70–80	60	158	Inguinal hernia repair	2
4	40–50	59	150	Breast tumor	2
**5**	**30–40**	**97**	**186**	**Cholecystectomy**	**1**
6	20–30	50	164	Hallux valgus	2
7	50–60	64	165	Arthroscopy	2
8	40–50	65	178	Hysterectomy	2
9	20–30	76	178	Sacrococcygeal cyst	2
10	30–40	78	175	Varicocele	1
11	50–60	69	158	Ureteroscopy	2
12	40–50	70	172	Pseudarthrosis	1
13	70–80	79	172	Circumcision	2
14	40–50	57	176	Fibroscopy/Colonoscopy	1
15	20–30	63	162	Hysteroscopy	1
**16**	**70–80**	**106**	**163**	**Coloscopy**	**3**
**17**	**60–70**	**74**	**175**	**Inguinal hernia repair**	**1**
18	30–40	60	168	Urinary lithiasis	2
**19**	**20–30**	**85**	**185**	**Mastectomy**	**1**
20	50–60	76	176	Colonoscopy	2
21	70–80	75	169	Hysteroscopy	2
**22**	**70–80**	**90**	**172**	**Urinary lithiasis**	**2**
23	20–30	60	183	Hydrocele	1
**24**	**30–40**	**100**	**190**	**Knee arthrosis**	**1**
25	40–50	86	173	Circumcision	2
26	70–80	49	159	Ovariectomy by laparoscopy	2
27	40–50	60	165	Ureteral stenting	2
**28**	**50–60**	**70**	**170**	**Inguinal hernia repair**	**1**
**29**	**30–40**	**97**	**174**	**Exploratory Laparoscopy**	**2**
30	60–70	98	166	Cholecystectomy	2

*The eight patients kept for analysis are in bold*.

### 3.1. Channel Selection

**Figure 8:**
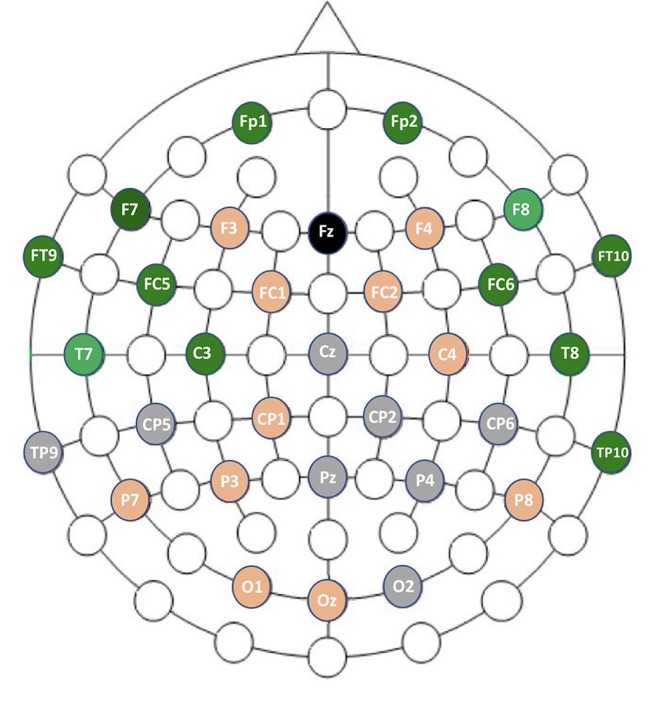
Graphic representation of channel performances. In gray are the faulty channels not included in the analysis. In orange are channels with mean AUCs ranging from 0.83 to 0.89 with GNB. In green are channels with mean AUCs ranging from 0.90 to 0.93 with GNB.

Channels marked as bad in two subjects or more were ignored. With the GNB, the three channels with the best mean AUCs and with a small standard deviation were F8 (AUC = 0.92 ±0.04), T7 (AUC = 0.91 ±0.04) and FC5 (AUC = 0.91 ±0.05). With the QDA, the three channels with the best mean AUCs and with a small standard deviation were F8 (AUC = 0.91 ±0.04), T7 (AUC = 0.9 ±0.04) and T8 (AUC = 0.89 ±0.06). All the values are reported in the Table 4 and results are displayed in the Figure 8. Channel F8 was the best regarding the two algorithms. Hence, we selected it as the best channel. The following computations have been done on the channel F8.

**Table 4:** Mean AUCs (± standard deviation) obtained for each channel with each algorithm (GNB and QDA).

	**GNB**		**QDA**	
**Channel names**	**AUC validation**	**AUC test**	**AUC validation**	**AUC test**
FP1	0.89 ± 0.06	0.89 ± 0.06	0.87 ± 0.07	0.87 ± 0.06
F3	0.85 ± 0.06	0.84 ± 0.07	0.85 ± 0.06	0.83 ± 0.06
F7	0.90 ± 0.05	0.88 ± 0.06	0.89 ± 0.05	0.87 ± 0.06
FT9	0.90 ± 0.05	0.87 ± 0.06	0.89 ± 0.05	0.86 ± 0.06
FC5	0.91 ± 0.05	0.89 ± 0.06	0.90 ± 0.05	0.88 ± 0.05
FC1	0.87 ± 0.09	0.86 ± 0.08	0.86 ± 0.1	0.84 ± 0.08
C3	0.90 ± 0.06	0.88 ± 0.06	0.89 ± 0.07	0.87 ± 0.07
T7	0.91 ± 0.04	0.88 ± 0.05	0.90 ± 0.04	0.87 ± 0.05
P3	0.89 ± 0.08	0.87 ± 0.08	0.86 ± 0.1	0.85 ± 0.09
P7	0.88 ± 0.07	0.85 ± 0.06	0.87 ± 0.07	0.85 ± 0.07
O1	0.86 ± 0.1	0.84 ± 0.07	0.81 ± 0.15	0.81 ± 0.11
Oz	0.87 ± 0.08	0.85 ± 0.07	0.83 ± 0.12	0.82 ± 0.09
P8	0.87 ± 0.07	0.84 ± 0.07	0.84 ± 0.12	0.82 ± 0.09
TP10	0.9 ± 0.06	0.88 ± 0.06	0.89 ± 0.07	0.87 ± 0.06
C4	0.89 ± 0.07	0.85 ± 0.08	0.88 ± 0.08	0.84 ± 0.08
T8	0.91 ± 0.05	0.88 ± 0.05	0.89 ± 0.06	0.87 ± 0.05
FT10	0.91 ± 0.05	0.89 ± 0.05	0.89 ± 0.07	0.88 ± 0.06
FC6	0.91 ± 0.06	0.88 ± 0.06	0.89 ± 0.06	0.86 ± 0.06
FC2	0.87 ± 0.08	0.84 ± 0.08	0.863 ± 0.09	0.83 ± 0.08
F4	0.84 ± 0.14	0.83 ± 0.1	0.83 ± 0.15	0.82 ± 0.1
F8	0.92 ± 0.04	0.9 ± 0.05	0.91 ± 0.04	0.89 ± 0.04
FP2	0.9 ± 0.05	0.89 ± 0.05	0.88 ± 0.06	0.87 ± 0.05

*The channels faulty on two patients or more were removed from the selection*.

### 3.2. Validation of the Selected Channel

**Figure 9:**
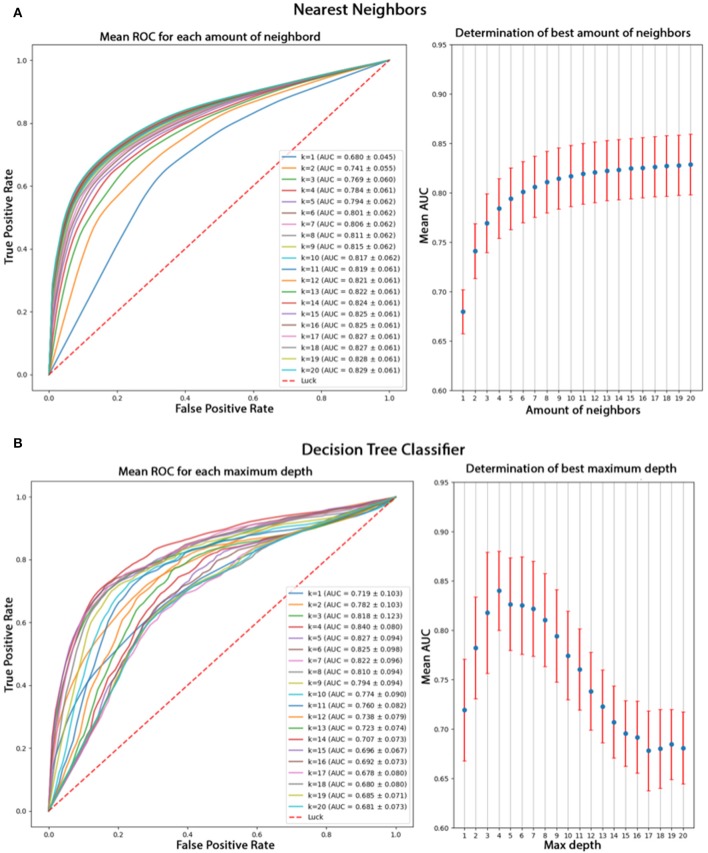
**(A)** Comparison of *k* values from 1 to 20 for the KNN algorithm based on their mean AUC after a cross-validation. The mean AUC seems to be growing with *k* and converging toward 0.84. **(B)** Comparison of different *k* values for DTL algorithm based on their mean AUC after cross-validation. The best AUC 0.84 is obtained for *k* = 4 and decreases for higher values.

**Figure 10:**
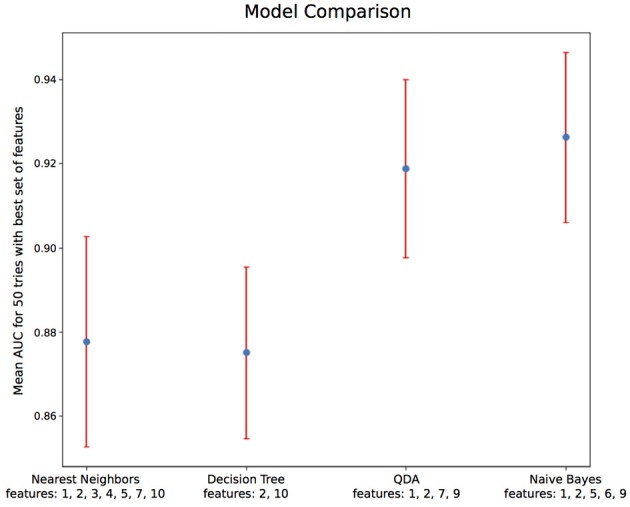
Algorithm comparison with optimal hyperparameters and feature sets. Among the 4 algorithms tested, Naive Bayes gives the best AUC of 0.92 ± 0.04. The features' numbers refer to the features listed in details in Table 2.

**Figure 11:**
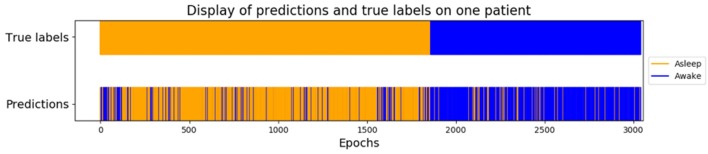
Example of prediction on one subject using the GNB method. Awake states before and after GA are concatenated at the beginning in orange, and GA epochs are at the end in blue, which means it is not a temporal representation but a classified one.

Using only features associated to channel F8, the goal was to classify epokes in Awake or Asleep using less variables possible. We compared the following algorithms: KNN, QDA, DTC, and GNB. While QDA and GNB do not have hyperparameters, KNN has the amount of neighbor and DTC has the maximum depth. To compare the best algorithms possible applied to our problem, we determined the hyperparameters accordingly with cross-validations. We separated our dataset in Training set (6 patients) and Test set (2 patients) leading to a total of 56 possible combinations. The mean AUC obtained on the Test set with all the combinations was used as a performance measure. We first computed the mean AUC for *k* neighbors ranging from 1 to 20 (Figure 9A). After computing very high *k* values (>100), the AUC seems to be converging toward 0.84. To limit computational time, we decided to take *k* = 20 for an AUC = 0.82±0.061 which is 0.98% of 0.84. Similarly, we computed the mean AUC for *k* maximum depths ranging from 1 to 20 (Figure 9B). The best AUC (0.84 ± 0.08) has been obtained for *k* = 4. Our optimal hyperparameters being fixed, we compared the four different algorithms by computing the mean AUC of every possible subset (512) of the 10 features used. We kept the best one obtained for each algorithm with their respective optimal features (Figure 10). The algorithm with the best AUC is the GNB with an AUC of 0.92 ± 0.04 using the features Standard Deviation, Sample Entropy, Power Spectrum Theta, Power Spectrum Alpha and Ratio PWS Theta/Beta. An example of prediction on one subject using the GNB method can be seen on Figure 11, with awake state on one side and the asleep state on the other side.

## 4. Discussion

We found that the best channel to monitor the DoA was F8, which corresponds to the frontal brain area. The temporal area, represented by the channel T7, also performed well whereas the central, parietal and posterior EEG channels poorly discriminated the states between Awake and Anesthesia. This result is in accordance with the use of EEG-based DoA monitors, such as BIS and Entropy monitors.

### 4.1. One Channel May Be Enough to Predict the Depth of Anesthesia

The location that gave the best AUC was the frontal channel F8 with a value of 0.92 ±0.04. This could even be improved with more complex features since the ones used here were based on the repartition of delta, theta, alpha, and beta powers in the EEG signals. Analysis was limited to four algorithms selected for their computational time, simplicity and familiarity. Other algorithms with optimal parameters may perform better than GNB. Also, using the physiological variables we recorded could be a source of improvement to discriminate the patient state. It would be interesting to establish a parallel between the present results with those of BIS, a commercially available EEG based monitoring system of the DoA. However because the EEG data processing and the algorithm at play are not in the public domain, it precludes comparison. Nevertheless, BIS does have several limitations including high inter-individual variations and low performance with volatile anesthetics (Whitlock et al., 2011), long latency and interferences with surgical knife and artifacts from movements or from forced air warming therapy (Hemmerling and Migneault, 2002). In addition it is not known if BIS can reduce the risk of intraoperative awareness (Avidan et al., 2011). As far as the clinical practice is concerned, less than 3% of the patients are monitored with an EEG during GA (Pandit et al., 2014), and when applied, it is a preprocessed EEG in the majority of the cases (Chander et al., 2014). Experimental studies published in the field of EEG during GA used between 2 and 128 EEG channels. To the best of our knowledge, there is currently no recommendation on the number of channels to use in an experimental protocol. The neuronal substrate of anesthesia remains to be understood. While the distribution of the anesthetics in the brain is thought to be homogeneous (Shyr et al., 1995), Uhrig et al. demonstrated that anesthesia disconnected frontal eye fields and premotor areas from parietal and cingular cortices (Uhrig et al., 2018). It could be related to the fact that monitoring the frontal area was sufficient to discriminate between Awake and Anesthesia. Finally, using the same methodology, Alotaiby et al. demonstrated that the use of 10–30% of the available channels was enough for emotion classification, motor imagery classification, and seizure detection (Alotaiby et al., 2015).

### 4.2. Potential Clinical Applications

Our result can have potential implications for clinical practice, as the minimal number of EEG channels needed to discriminate a proper DoA had not been previously studied. The observation of the typical alpha-delta pattern on a single channel appeared to be sufficient to discriminate between Awake and Anesthesia and thus to objectively assess the DoA. Only a few monitors have been developed and validated to assess the DoA in routine clinical use. Our results suggest that it could be useful to use a single EEG channel to improve the multimodal prediction of the DoA such as the one developed by Schneider et al. (2014). In addition, the prediction of the depth of sedation (DoS) in the ICU is even trickier than under GA. No monitor is currently available and this remains an unmet need for intensivists (Vincent et al., 2016). Indeed BIS is not appropriate due to the variety of drugs used and the number of muscular artifacts (Vivien et al., 2003). In that context, the present results pave the way to monitor the DoS by combining physiological parameters with a single channel EEG monitoring.

### 4.3. Limitations and Methodological Considerations

Our study has some limitations due to real life conditions. The impedance tolerated for the electrodes was higher than in laboratory studies due to subject's different skull anatomies and sometimes important hair density impeding good signal transmission. Despite this hindrance, our spectrograms displayed good recording quality. However, to ensure that the data, which were used for the ML algorithm, were not corrupted by any artifact and had a low level of noise, we excluded 22 patients out of the 30 included. This small sample may be the main limitation of our work, however we used the best suited ML protocols (cross-validation). A larger sample of data should be used to confirm the present results. Our anesthesia protocol was limited to propofol/sufentanil induction and maintenance with sevoflurane/sufentanil. Therefore, we cannot state that our conclusions would stand with other anesthetics. However, based on the recent literature and the strong alpha power in the frontal lobes, it seems reasonable to think that extrapolation would be possible. Moreover, the results could be improved thanks to a larger set of features and algorithms. We applied a binary classification; thus, it would be interesting to compare our algorithm with a BIS-driven DoA using a single EEG channel. Indeed, patient's states were binary classified between awake and asleep, which may lead to errors around LOC and ROC where the separation between awake and asleep is less clear. In these states some features may not be directly related to physiological variations but to environmental stimuli such as the head pressure on the cap. While such factors cannot be discriminated by the algorithms, we are confident that our results have not been biased by these phenomena since LOC and ROC were confirmed clinically and electrophysiologically and the data collected during these states were not used to feed the database.

## 5. Conclusion

The purpose of this study was to determine the best channel to predict the DoA among the 32 EEG channels we recorded. We found that channel F8 gives the most valuable information on single-channel monitoring. More generally, the fronto-temporal region gives good results to monitor GA with a single channel. Our results are consistent with the current use of DoA monitors and with the previous studies demonstrating that anteriorization and high amplitude slow-waves gave decisive information on the patient's vigilance states.

## Ethics Statement

The study protocol has been approved by Pr. JE Bazin, head of the ethics committee of the French society of anesthesiology (SFAR) under the number IRB 00010254-2016-018.

## Author Contributions

CD, PH, and P-PV conceived of and designed the study. CD and AB acquired EEG data. PH, NV, and AB analyzed data. CD, PH, AB, J-PT, and P-PV interpreted the data. CD and PH wrote the manuscript. All authors contributed to critical review of the manuscript.

### Conflict of Interest

The authors declare that the research was conducted in the absence of any commercial or financial relationships that could be construed as a potential conflict of interest.
